# Exploring the historical distribution of *Dracaena cinnabari* using ethnobotanical knowledge on Socotra Island, Yemen

**DOI:** 10.1186/s13002-021-00452-1

**Published:** 2021-04-01

**Authors:** Abdulraqeb Al-Okaishi

**Affiliations:** Department of Forest Botany, Dendrology and Geobiocoenology, Faculty of Forestry and Wood Technology, Mendel University in Brno, Zemědělská 3, 613 00 Brno, Czech Republic

**Keywords:** Ethnobotany, Toponymy, Phytotoponym, Socotra Island, Dum al-akhawin, Dragon’s Blood Tree

## Abstract

**Background:**

In this study, we present and analyze toponyms referring to Socotra Island’s endemic dragon’s blood tree (*Dracaena cinnabari*) in four areas on the Socotra Archipelago UNESCO World Heritage site (Republic of Yemen). The motivation is the understanding of the past distribution of *D. cinnabari* trees which is an important part of conservation efforts by using ethnobotanical data. We assumed that dragon’s blood trees had a wider distribution on Socotra Island in the past.

**Methods:**

This research was based on field surveys and interviews with the indigenous people. The place names (toponyms) were recorded in both Arabic and the indigenous Socotri language. We grouped all toponyms into five different categories according to the main descriptor: terrain, human, plant, water, and NA (unknown). Also, this study identified current and historical Arabic names of dragon’s blood trees of the genus *Dracaena* through literature review.

**Results:**

A total of 301 toponyms were recorded from the four study areas in Socotra Island. Among names related to plants, we could attribute toponyms to nine different plants species, of which six toponyms referred to the *D. cinnabari* tree, representing 14.63% of the total phytotoponyms in the category. Three historical naming periods prior to 2000 could be identified. The most commonly used name for dragon’s blood trees (*D. cinnabari*, *D. serrulata*, *D. ombet*) appears to be “*ahrieb*” “إعريهب” and its resin “*dum al-akhawin*” “دم الأخوين,” while derived (mixed-cooked) products are called “*eda’a*” “إيدع,” while regionally different names can be found.

**Conclusion:**

The place names that refer to *D. cinnabari* are herein suggested to represent remnant areas of once large populations. Therefore, the toponyms may support known hypotheses based on climate models that *D. cinnabari* had a wider distribution on Socotra Island in the past. This study also confirmed the historical importance of dragon’s blood.

## Background

### Ethnobotany and toponymy

Since the beginning of civilization, people have used plants for food and medicine, as well as materials for construction and the manufacture of crafts and many other products [1]. In addition, plants have extensive symbolic uses, such as in art, mythology, and literature [2]. Interactions between people and plants have accumulated large bodies of traditional ecological knowledge built by a group of people through generations living in close contact with nature. It includes a system of management of resources, classification, and observations [3]. The term *ethnobotany* was designated by Harshberger [4], originally linked to the description of plant materials use by Aboriginal Australians. Ethnobotany later became a more ecological term, focusing on relationships, interrelationships, and interaction with a cultural perspective [5]. Harshberger [4] considered that ethnobotany could also help in studying the past distribution of plants.

People need to give names to areas to label, identify, and locate them in space [6]. When the indigenous inhabitants give such names, they often use them for distinctive spatial orientation, landscape features, natural phenomena, fauna, flora, natural substances, and names of tribes or important individuals [7]. Toponyms are conservative, and although the language and wording may evolve, the roots of place names are not likely to be altered by changes in human activities in the landscape through time [8–10]. Toponyms can be easy to record and may provide useful information about the history of a land and its resources [11]. According to Berkes [12], systematic meaning through toponyms, oral history, and spiritual relationships form part of a dimension of traditional ecological knowledge. Place names may also reflect intensity of land use, the extent of traditional ecological knowledge and population density of the associated society [13], historical-cultural environmental development [14], settlement history [15], and archaeology [16]. They can be used for studying current environmental issues such as tracking recent climate changes and perceptions of those changes [17–19], water issues [20], and the climatic environment [21]. The systematic study of indigenous place names can be an approach to the mapping of ethnoecological knowledge and understanding of the evolution of the landscape [22]. Toponyms concerning plants (phytotoponyms) and animals (zootoponyms), named according to what people used to see in their everyday life, can be the indicators of the present, or former, presence of certain species [23–26].

Phytotoponyms may provide information on spatial locations, temporal information, and landscape [27]. They have been used to study landscape ecology and botany [10], vegetation cover, and long-term vegetation degradation [28]. According to Cunningham et al. [11], local knowledge may sometimes be the only evidence that remains where some plant species used to occur. Phytotoponyms, not just the common plant names, also describe the usage of the species as food, medicine, fabric, or other activities [29, 30] and their interaction with the surrounding environment [31, 32]. Therefore, these specific types of place names can be used for the reconstruction of past events, specific vegetation, or certain species [10, 33–38].

Socotra Island, the largest island of the Socotra Archipelago (Yemen), located at the crossroads between the Red Sea, the Arabian Sea, and the Indian Ocean, was recognized as a regional center of biodiversity. The archipelago is not only rich in biodiversity with spectacular endemic species, with more than 37% of endemic plant species [39], but also rich in traditions that conserve this biodiversity until today. The landscape changes over the last centuries and/or millennia have rarely been studied. Paleoclimate studies indicate wetter periods in the Holocene on Socotra [40, 41]. However, there is very little paleontological or data available for the reconstruction of historic and prehistoric landscapes on the island [42]. In the more recent past (decades to a century), landscape changes were investigated with relation to vegetation, using historical photographs [43], a combination of old aerial photographs, satellite images, and repeated field measurements to study changes in population of *Dracaena cinnabari* and *Boswellia elongata* [44]. More recently, Rezende et al. [45] studied land productivity on Socotra using NDVI derived from satellite images in the last 20 years, showing a highly dynamic system.

The current Socotra landscape is the witness of dynamic changes in the past. It was subjected to the centuries of human land-use pressures. One of the most important was resin (incense, myrrh, dragon’s blood) harvesting, resulting in the numerous wounds on dragon’s blood trees [46] and the decline of dragon’s blood trees’ distribution [47]. The stone walls that cover large areas of the Socotra landscape refer back to the intensive management system of dragon’s blood, frankincense, and aloe for which Socotra was famous [48]. Currently, Socotra faces the effect of overgrazing [49], climate change [44], and unsustainable harvesting of dragon’s blood [46]. This research contributed to Socotra nature conservation, focusing on dragon’s blood trees’ distribution in the past using traditional environmental knowledge. This research is one of the first in the region which uses toponyms to explore the past environment and vegetation and the first research of its kind on Socotra. It is considered a pioneering research that will pave the way for other researchers. This research participates in the documentation of the Socotra traditional knowledge and its language.

### Socotra and dragon’s blood

The genus Dracaena is classified in the family Asparagaceae subfamily Nolinoideae (The Linnean Society of London 2016). *Dracaena cinnabari* balf.f Socotra dragon’s blood tree belongs to the dragon’s tree group which contains 14 species as reviewed by Maděra et al. [50] based on Marrero et al. [51] and Marrero [52]. The Socotra dragon’s blood tree (*Dracaena cinnabari*) is a flagship species of Socotra [53, 54]. It was a very important tree in ancient times due to a historically highly prized product called dragon’s blood, a red resin extracted for a wide range of uses including coloring and local medicine [39, 50]. Some believe that the name Socotra could even be derived from “Sukkatira” or contracted from “suq qatra,” where suq is the Arabic word for “market” and qatra for “dragon’s blood,” which means “drop” related to the dropping of the liquid resin pieces from the stem of the plant before drying it [55, 56]. The first who mentioned *D. cinnabari* resin was the unknown author of the Periplus of the Erythrean Sea around the mid-first century ad, who called it “cinnabar” [57]. Dioscorides (90 ad) mentioned the resin in his book “On Medical Material” as Kinnabari “cinnabari,” brought from Africa [58].

Names of dragon’s blood tree and its resin have been recorded by old Arabic literature [59–63], by researchers who visited Socotra [55, 64–67], and recently by [39, 46, 68, 69].

Several local names for *Dracaena* may indicate the significance of the ethnobotanical knowledge as an important source of information that can be used for tracking the history of these names or link them to the land by studying place names (toponyms). The aim of this study is to use this ethnobotanical knowledge to explore the spatial distribution of toponyms related to *Dracaena cinnabari* tree and its potential as an information source to assess the past distribution of this unique flagship species on Socotra Island.

## Material and methods

### Study area

Socotra Island is part of the unique Socotra Archipelago natural UNESCO World Heritage Site (Republic of Yemen), with a total area of 3,675 km^2^ [70–72]. Livestock grazing, fishing, agriculture (mainly date palm plantation), and collection of non-timber forest products are the main activities of people; the latter includes gathering resins such as frankincense, myrrh, and dragon’s blood and harvesting of aloe juice [39]. Socotra was famous for these products in ancient times [39]. There are approximately 100 thousand inhabitants currently estimated, most of them live in coastal areas especially in the main cities of Hadibu and Qalansia. The main language is Socotri which is one of the Semitic languages [73], and Arabic is the official and commonly spoken language. Socotra Archipelago is recognized as a regional center of biodiversity, announced as a UNESCO World Natural Heritage Site since 2008 [71]. The Socotri people live in a relatively isolated area and are strongly connected to their land. Socotra is approximately 350 km far from the nearest mainland Yemen and in the past has been reached only by ships, being disconnected from the mainland during the monsoon months that makes the island more isolated till the opening of Socotra airport in 2000, which opened Socotra to the outside world [74].

### Study areas selection

**Fig. 1:**
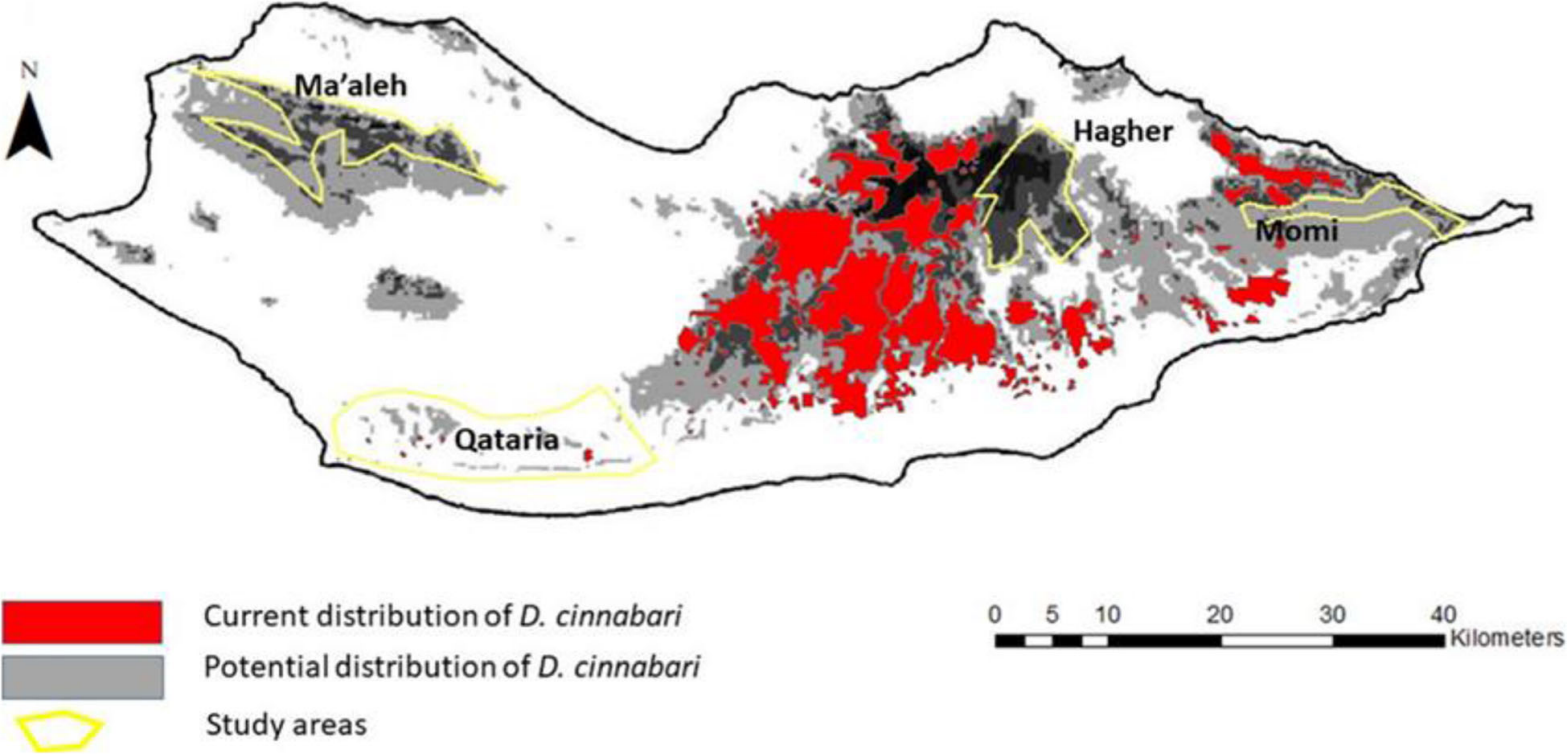
Map showing the study areas (Hagher, Momi, Qatanin, Ma’aleh) in integrating two maps with the current and potential distribution of *D. cinnabari* according to Maděra et al. [54] and Attorre et al. [47], respectively

Four areas have been selected by overlapping of two maps belonging to different datasets. The first map contains the current distribution of *D. cinnabari* [54], and the second map is the potential distribution of *D. cinnabari* according to its ecology [47]. The areas of the potential distribution not overlapped by the current distribution were selected for the study (Fig. 1).

### Data collection

#### Survey technique and toponym meaning

Fieldwork was carried out by visiting the areas and interviewing people residing in the area. The place names were collected, and the meaning was directly obtained on the spot in collaboration with a local guide. The meaning of the names was discussed in detail with the indigenous people. The positions of the places have been recorded as possible by GPS. During the fieldwork, three types of data were recorded: (1) toponyms, (2) visual observation of existing *D. cinnabari* trees in nature, and (3) interview with the people about the area, in particular, the occurrence of *D. cinnabari* in the area. Three areas were visited personally (Hagher, Momi, Qataria), and due to logistic limitations, the place names for the fourth area (Ma’aleh) were recorded remotely through communication with local people. The place names were recorded by fieldwork and remotely further confirmed by sending them to the other two residents to ensure the meaning. Local people also have been asked if there are any names related to the dragon’s blood tree and its distribution. GIS ArcMap was used to plot the georeferenced toponyms for three areas (Hagher, Momi, and Qataria) and those from Ma’aleh by approximation. A detailed literature review of Arabic and Western sources was carried out to investigate current and old names for the dragon’s blood tree and its resin. Somali names for *D. ombet* were collected by direct communication with Mr. Ahmed Ibrahim Awale, and the same for Sudani names for *D. ombet* by indirect communication with Dr. Iqbal Madani.

## Results

### Tracking dragon’s blood names through history

**Fig. 2:**
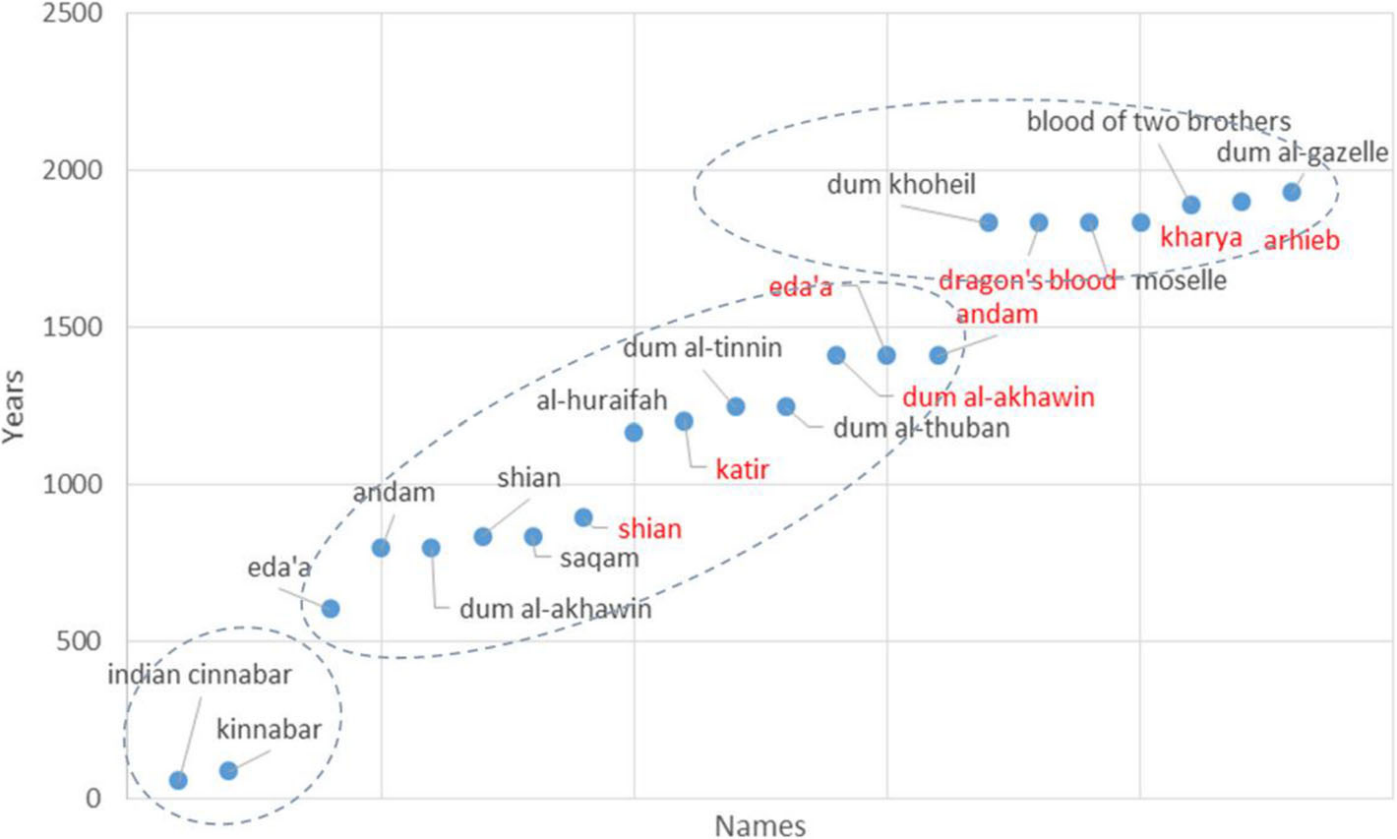
Different names of dragon’s blood resin and tree (written in red) as derived from the literature (first century ad–2000 ad). The literature list and the years listed in Table 1

From literature, we can distinguish three naming periods (Fig. 2). A variety of names for dragon’s blood appear during the golden era in science in the Islamic Arabic world (ca. 800–ca. 1500 ad). The last period represents the new western renaissance and scientific exploring missions, especially from Europe. The described period in this study begins in the year 60 ad, with the appearance of the first name referred to dragon’s blood, and ends in the year 2000 with the opening of Socotra to the outside world—the opening of Socotra International Airport. The horizontal oval shape shows that the naming was at close intervals, while the oblique oval shape indicates that the naming appeared at long intervals (Tables 1 and 2).

**Table 1:** Appearing of dragon’s blood names (resin/tree) from the first century ad to 2000 ad

No.	Author	Referred to	Dragon’s blood name	Year	Notes
1	Breasted [57]	Marchant	Indian cinnabar	60	Resin
2	Breasted [57]	Dioscorides	Kinnabar	90	Resin
3	Abu Hanifa [60]	Al-sulaik	Eda’a	605	Resin
4	Ibn Manzur and Mukarram [61]	Al-asmai	Andam	800	Resin
5	Ibn Manzur and Mukarram [61]	Al-asmai	Dum al-akhawin	800	Resin
6	Ibn Sallam [59]		Shian	838	Resin
7	Ibn Sallam [59]		Baqam	838	Resin
8	Abu Hanifa [60]		Shian	869	Tree
9	Ibn Manzur and Mukarram [61]	Ibn Barii	Al-huraifah	1165	Tree
10	Abi Umran [75]		Katir	1204	Resin
11	Ibn Al-baitar [76]		Dum al-tinnin	1248	Resin
12	Ibn Al-baitar [76]		Dum al-thuban	1248	Resin
13	Al-Firuzabadi [62]		Dum al-akhawin	1410	Tree/resin
14	Al-Firuzabadi [62]		Eda’a	1410	Tree/resin
15	Al-Firuzabadi [62]		Andam	1410	Tree/resin
16	Wellsted [64]		Dum khoheil	1835	Resin
17	Wellsted [64]		Dragon’s blood	1835	Tree/resin
18	Wellsted [64]		Moselle	1835	Resin
19	Balfour [65]	B.C.S	Kharya	1835	Tree
20	Breasted [57]	Bent	Blood of two brothers	1893	Resin
21	Forbes [55]		Arhieb	1899	Tree

**Table 2:** Dragon’s blood names (resin/tree) frequency from the first century ad to 2000 ad

No.	Name	Frequency			Authors
		R	T	T/R	
1	Cinnabar	3			[57, 77]
2	Eda’a	9	1	1	[59–64, 75, 78–82]
3	Andam	5	1	1	[59, 61–63, 76, 80, 83]
4	Dum al-akhawin	19	2	1	[59, 61–63, 75, 76, 78–90]
5	Shian	7	2		[59, 63, 75, 76, 79–82, 90]
6	Baqam	1			[59]
7	Katir	4	1		[63, 66, 75, 78, 87]
8	Al-huraifah		1		[61]
9	Dum al-tinnin	1	1		[63, 76]
10	Dum al-thuaban	3	1		[63, 76, 80, 82]
11	Dum khoheil	1			[64]
12	Dragon’s blood	4	2	2	[55, 57, 64, 65, 77]
13	Moselle	1			[64]
14	Kharya		1		[64]
15	Blood of two brothers				[57]
16	Arhieb		1		[55]
17	Dum al-gazelle		1		[63]

**Fig. 3:**
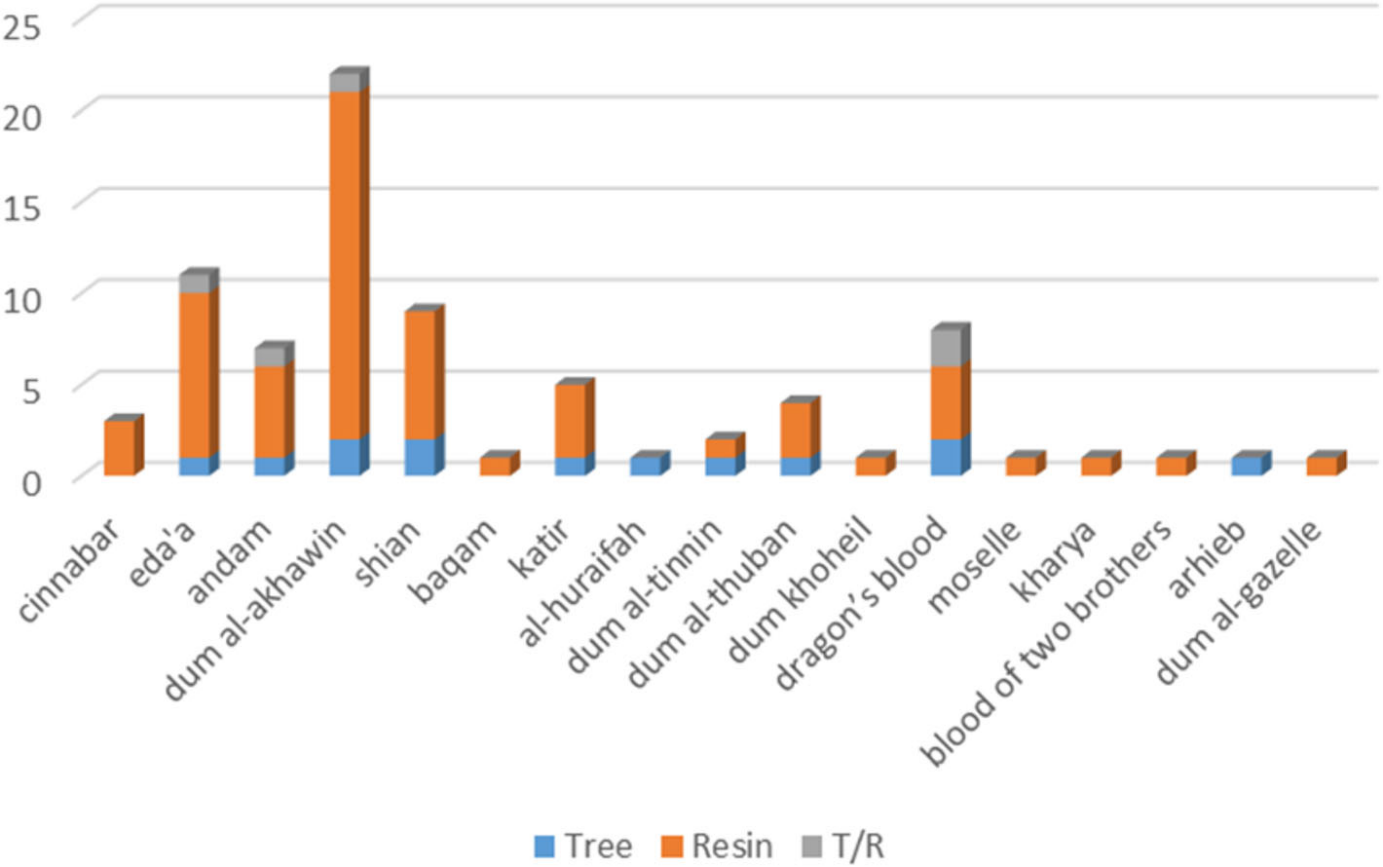
Frequency of appearing of dragon’s blood (tree and resin) names in literature from 1st century ad to 2000 ad, T/R means that the name is given to both the tree and the resin, references listed in Table 2

Depending on the number of sources for each time, the frequency of names can be limited (e.g., few first-century ad sources). High-frequency words are the occurrences of eda’a, dum al-akhawin, and dragon’s blood; medium frequency words are cinnabar, andam, and katir; other names are in low frequency. Most of the names referred to the resin, and a few referred to the tree (shian, al-huraifah, and kharya). The two names for the resin appearing in the first period (Fig. 3) were treated as one name because they came from the same origin “cinnabar.” Four common names for the dragon’s blood tree appear to be “dum al-akhawin,” “eda’a,” “al-huraifah,” and “shian,” besides the English name “Dragon’s Blood Tree,” of course.

The contemporary names (Table 3) were written according to where they occur in the references; names from Yemen, Saudi Arabia, and Sudan were written in simplified English by the author. The names representing four species of *Dracaena* distributed in the Arab World are presented in five languages (Socotri, Arabic, Hadandawa, Somali, and Amazigh). All names in the table are for dragon’s blood tree, and the names of the resin “emszoloh” and “iydiha” are added from Socotri. The word “dum al-akhawin” is used as a name for the tree and the resin.

**Table 3:** Contemporary names of dragon’s blood (tree/resin) in the Arabic region [39, 46, 68, 69, 91]

Area (***D.*** species)	Dragon’s blood names (tree/resin)					
Socotra (*D. cinnabar*i) [39, 46]	A’arhiyib	Iydiha^a^	Emzoloh^a^	Ahrieb	Dum al-akhawin	
Yemen (*D. serrulata*) [68]	Airob	Kasar	Kasl	Arrab	Khwas	Faliqat al-gawz
Saudia (*D. serrulata*) [69]	Arab	Khazm	Khazami	Arrab	Khaws	Azaf
Oman (*D. serrulata*) [91]	Areeb			Ariab		Ayrob
Sudan (*D. ombet*)^b^	Embet	Emet	Ras al-shitan	Shagart al-Tinnin		
Somalia (*D. ombet*)^b^	Dinaw	Mooli				
Morocco (*D. draco)*^b^	Ajgal					

^a^Dragon’s blood resin

^b^By communication (see data collection)

### Toponyms

A total of 301 toponyms were recorded from the four study areas in Socotra Island (Table 4), which characterize how the Socotri people view their landscape. Toponyms were clustered in six broad categories based on their meaning:

1. Animal: place name referring to animals such as livestock, birds, other
2. Human: place names referring to human body parts, names, feeling, interaction, tools
3. NA: place name with unknown meaning
4. Plants: place names referring to plant species, density
5. Terrain: place names referring to the shape and color of the landscape
6. Water: place names referring to water such as rain, streams

**Table 4:** Distribution of the toponyms among categories and areas

Category/area	Hagher	Ma’aleh	Momi	Qataria	Total
**Animal**	7	8	6	5	26
**Human**	26	15	17	15	73
**NA**	9	7	10	6	32
**Plant**	17	8	6	10	41
**Terrain**	43	21	24	25	113
**Water**	7	0	6	3	22
**Total**	109	59	69	64	301

Most toponyms were recorded from the Hagher, and the lowest number were recorded from Ma’aleh as this was through indirect communication.

**Fig. 4:**
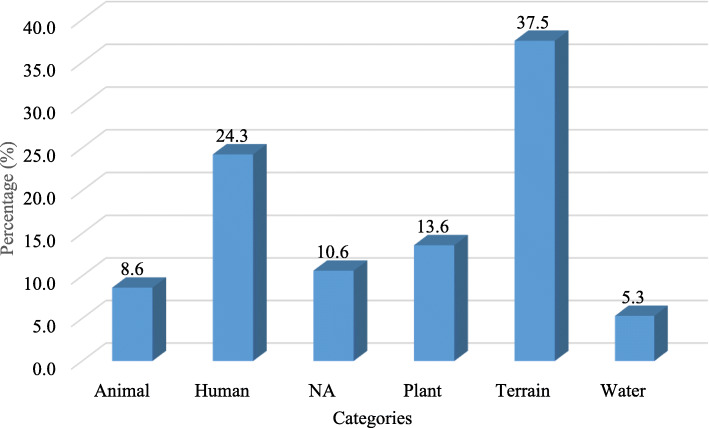
Frequency of toponym categories in the four selected areas on Socotra, summary of 301 names

**Fig. 5:**
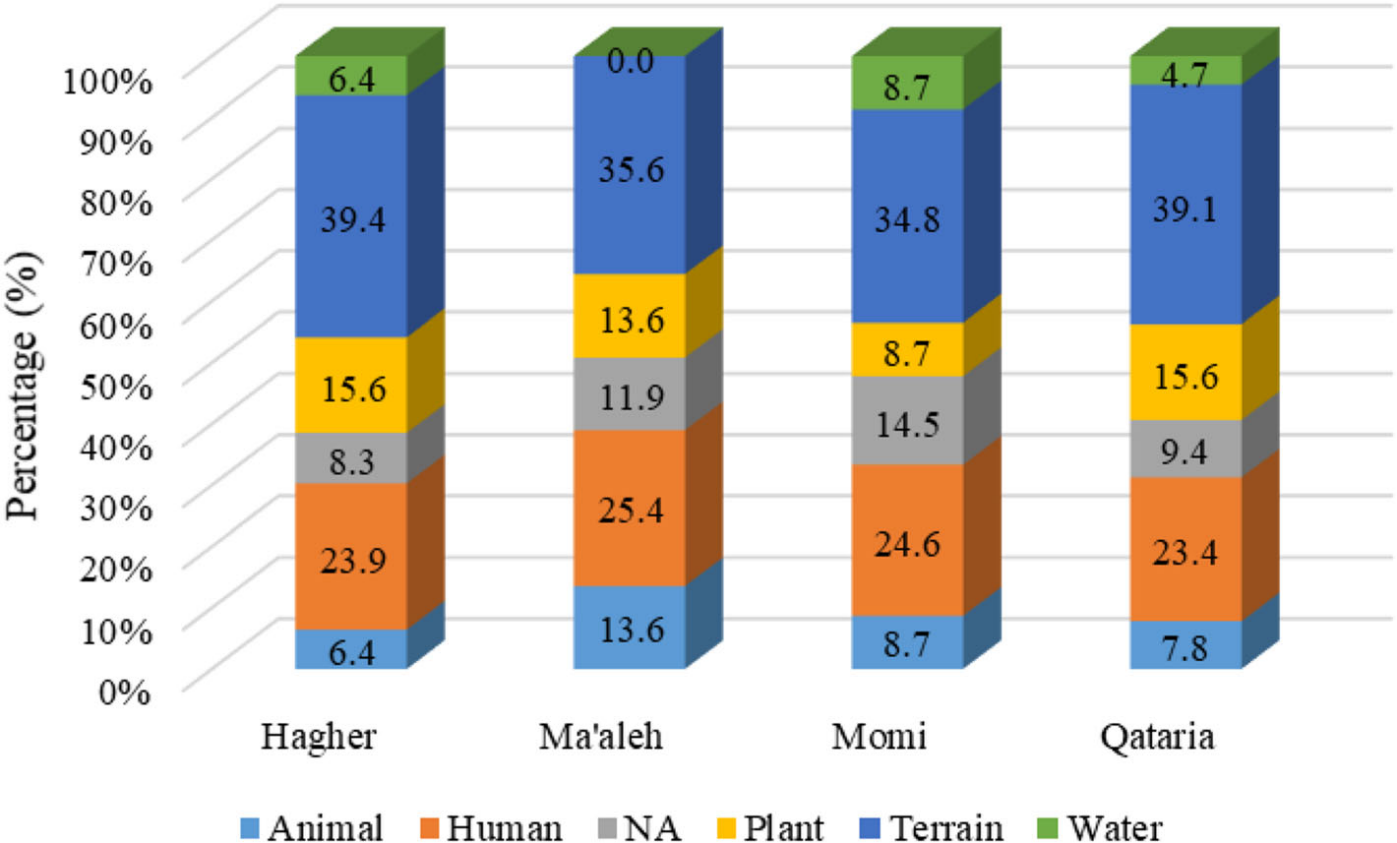
Frequency of toponym categories within each area

It is clearly visible (Fig. 4) that the most represented toponyms were related to terrain (37.5%). People not only describe the topography of the land such as mountain, hill, flat, and rock but also describe the general view of those areas such as open, narrow, protected, high, and low; the people also describe the soil and color of the land. Terrain toponyms followed by names referring to human characters and activities (24.3%). Interestingly, most of these names referred to human activities such as playing, jumping, relaxing, or giving, and that can be related to a once frequent activity in the place, yet they also use feelings such as hunger and fear and parts of the body like the ears, neck, and teeth if they resembled the topography by human parts. There are no naming places after people except two names for the tribes. The plant names represent 13.6%, varying between 8.7 and 15.6% among study areas (Fig. 5). These three categories have a higher percentage within all study areas (Fig. 5). Toponyms related to animals and water have a lower percentage of 9% and 6.9%, respectively, and there are names of unknown meaning (10.6%).

Given the importance of plants for people in Socotra, it is not surprising that the names referred to plants come in the third position. Based on further analysis, we divided the plant names into five subcategories (Table 5) based on their meaning;

1. Unidentified: place names referring to the unidentified plant species
2. Identified: place names referring to the identified plant species such as the Socotri word “Tayf” for Aloe
3. General: place names referring to the word “plant” without any specification
4. Density: place names referring to the plant density
5. Grass: place names referring to grassland

**Table 5:** The frequency of place names in individual sub-categories of the phytotoponyms

Sub-category	Unidentified	Identified	General	Density	Grass
Number	5	22	6	6	2
Percentage (%)	12.2	53.7	14.6	14.6	4.9

**Fig. 6:**
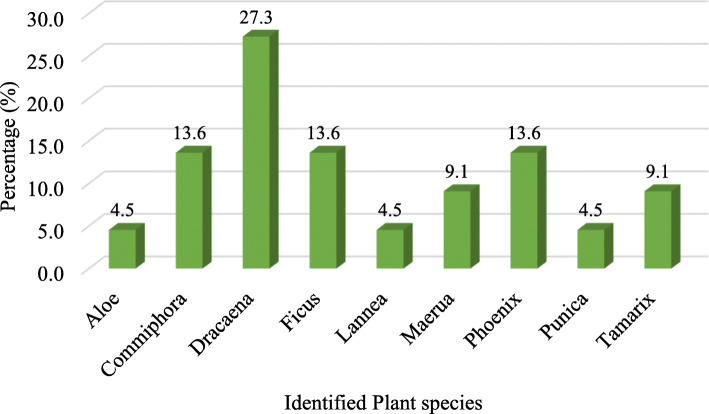
Frequency of phytotoponyms in the subcategory referring to identified plants (genus/species), including *Dracaena cinnabari* (27.3% of the subcategory)

**Fig. 7:**
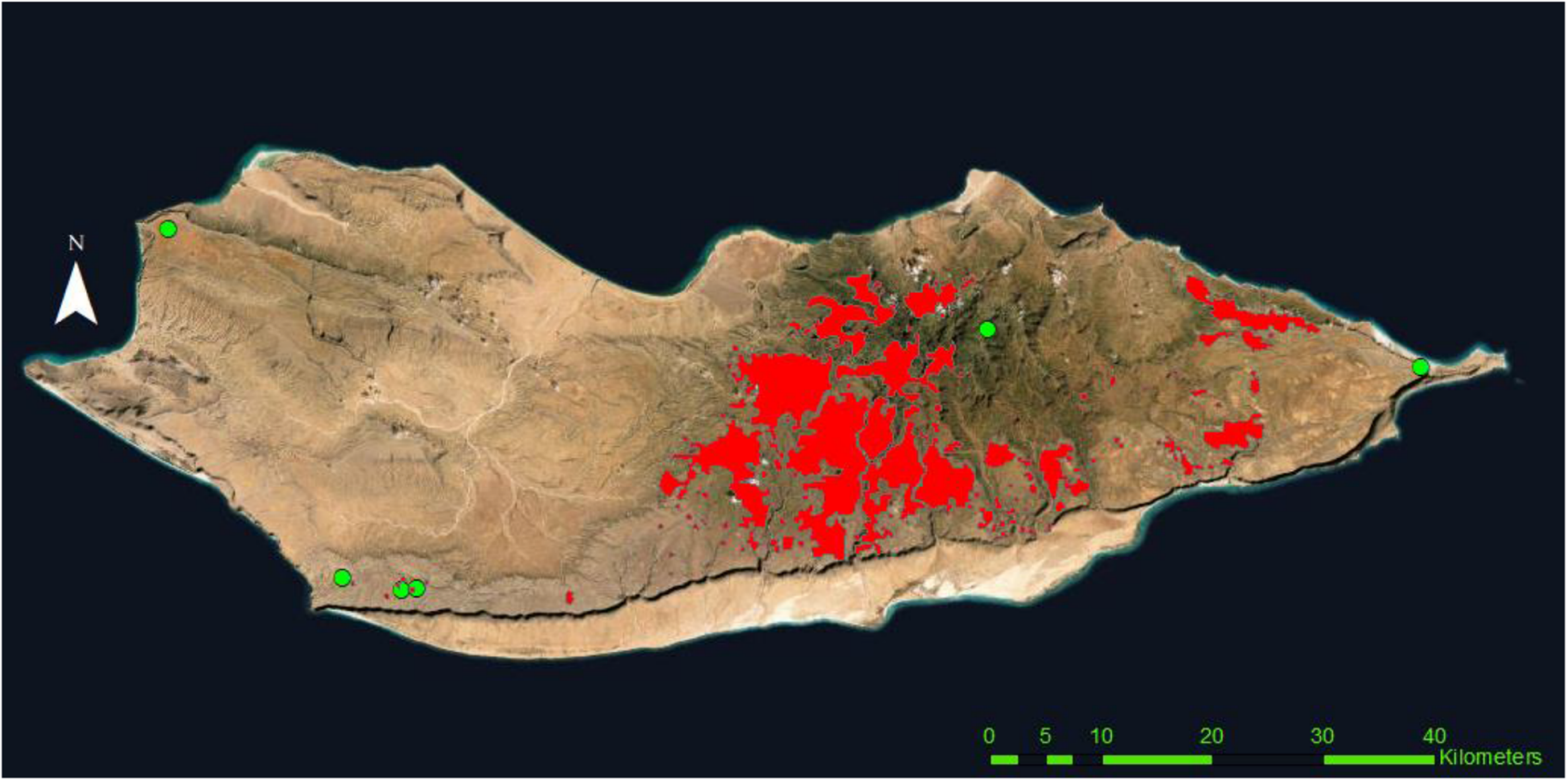
The map of the distribution of the toponyms related to the *D. cinnabari* tree (green circles) in Socotra Island, in red, the current distribution of *D. cinnabari* by Maděra et al. [54]

We found six records of phytotoponyms related to *D. cinnabari*, which represent 2% from all toponyms recorded, 14.6% from the phytotoponyms, and 27.3% from the subcategory of phytotoponyms referring to identified plant species (Figs. 6 and 7).

### Reconstruction of dragon’s blood tree distribution

**Fig. 8:**
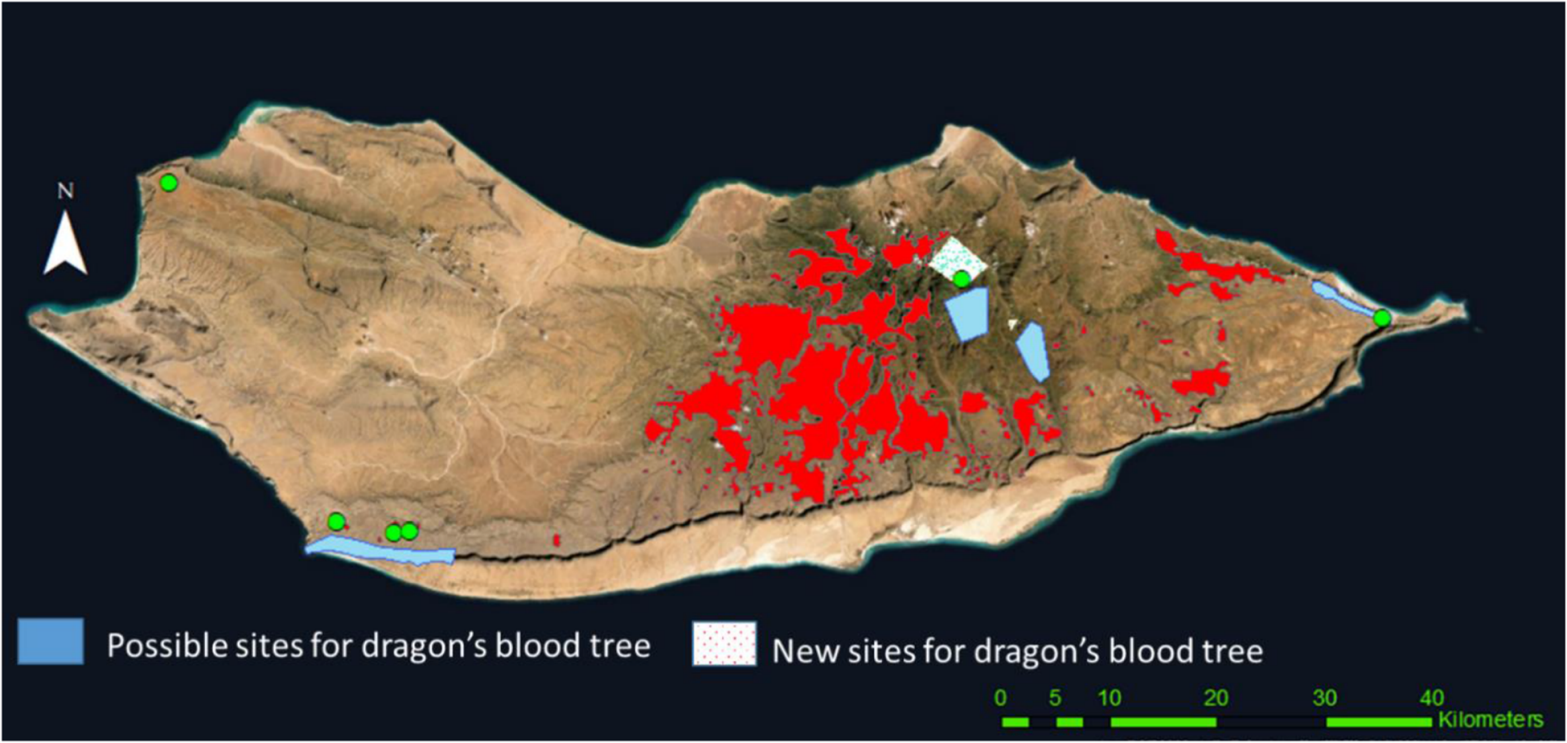
Map of new sites of dragon’s blood tree from the fieldwork (white polygon with dots), not published by Maděra et al. [54] (red color), and possible sites for dragon’s blood tree according to the local community for further field research (blue polygons), toponyms related to *D. cinnabari* (green points)

Among the six phytotoponyms referring to *Dracaena* (Fig. 7), there is one located in the far end of Ma’aleh Mountains and two on the eastern edge of Momi plateau, and both far from the currently known *D. cinnabari* distribution. The other toponyms include three localities in Qataria where few remaining trees are known (Maděra et al. [54]) and one in the Hagher, at the border of the current distribution of *D. cinnabari*. People in those areas have been interviewed individually, within-group discussion and communication, and the results are presented in the map (Fig. 8). The people from Hagher speak about possible sites for *D. cinnabari* close to the areas of current distribution, and people in Qataria and Momi speak about the possible sites for *D. cinnabari* in the cliffs towards the sea; however, we could not confirm this during the study visit. During the fieldwork, we recorded new sites with *D. cinnabari* (Fig. 8) where the trees have been observed. Both possible and new sites need further research.

## Discussion

### Tracking dragon’s blood tree phytotoponyms

#### Names through history

According to the estimates based on genetic research, Socotra was inhabited ca. 6000 years bp [92]. From the old manuscripts, cinnabar was derived from the ancient Greek name for red mineral (mercury sulfide HgS) and adapted as the scientific name for Socotra dragon’s blood tree (*Dracaena cinnabari*) and has no relation to current or historical Arabic names only perhaps by color. Arabic literature [59, 61, 84, 86] use dum al-akhwin as a common Arabic name for dragon’s blood resin, sometimes for the tree without specification of the sources and for the resin brought from Socotra, and this name continued to be used from the past until today. “Eda’a” is the only local Socotri name that appears significantly in Arabic literature [60, 87] especially for the resin of dragon’s blood tree due to its famous use in medicine. Other Arabic old names for dragon’s blood such as “andam” [76, 83], “dum al-thuban” [76], and “shian” [79] are also used for other products, as an example, “andam” used for logwood. “Dum al-tinnin” [76] is the Arabic translation of dragon’s blood, and “katir” is the general name for drops. The first record for the local name of dragon’s blood tree was in 1899 by Forbes [55], but *eda’a* and *emsello* (“moselle”) have been mentioned also by Wellsted [64]. Cabo González and Bustamante Costa [93] suggested that there is a weakness in dictionaries and confusion of terminology related to dragon’s blood names and gave an example of “andam” and “baqam.” However, *andam* with its red color can bring some confusion but *baqam* before 1500 ad was rarely mentioned in Arabic literature, in my review just once by Ibn Manzur and Mukarram [61]. From their reviews, “shian” is a popular name for dragon’s blood in Morocco which is of Persian origin; however, the translation of dragon’s blood to Persian is “khun-siawshan” which appears in 1205 by Abi Umran [75], who divided the names by area: Arabia “dum al-akhawin and eda’a,” Morocco “shian,” and Persia “khun-siawshan.” In their review, three other names of dragon’s blood appear “Itr mansham,” “Hagun,” and “Tabdigha” referenced to Abu al-khair (ca 1200) [80], and according to them, “Tabdiga” is from the Amazigh language.

There is no sign of loss of cultural knowledge as a consequence of Arabic intervention. Only one name appears from Arabic (name of the plant) and that could be from an individual perspective or newly named, but there are still names that cannot be explained by the local people and that show their ancient roots. Although a large part of Socotra is uninhabited intensively by people—around 100 thousand inhabitants in 3625 km^2^, many spaces have no urbanization. The human interaction is very clear: by giving names for each patch of land (personal observation), this confirms that the Socotri people have strong knowledge, understanding, interaction, and connection with places, and the huge walls “eggehon” dominating the landscape especially in the higher altitudes has been claimed as circumstantial evidence that the wall system on Socotra might be a sign of past historical intensive farming activities for incense, dragon’s blood, or aloes [48].

#### Current names

There are four dragon’s blood trees in the Arabic-speaking regions, *D. cinnabari* in Socotra; *D. serrulata* in Yemen, Oman, and Saudi Arabia; *D. ombet* in Saudi Arabia, Sudan, Egypt, Ethiopia, and Somalia; and *D. draco* subsp. *ajgal* in Morroco. We have seven groups of names according to the area (Table 2). In my opinion, “Ahrieb” with its different form of writing and pronunciation is the common local name for all dragon’s blood tree species in Arabic region (Yemen, Socotra, Oman, and Saudia Arabia) (Table 3) [39, 46, 68, 69, 91]. Different names appear such as “Ajgal” in Morocco in the Amazigh language, “Embet” in Sudan in the Hadandawa language, and “Mooli” in Somalia in the Somali language. In Socotra, there is only one local name for the dragon’s blood tree which is “ahrieb;” other names such as “emsello” is for the pure product or “eda’a” is the mixed product with tree bark [39, 46, 68], and due to the difference in dialect, people of western Socotra call it “ahrieb,” with “ح” instead of “ع”. Other current Arabic local names linked the leaves and their similarity with palm leaves such as “khwas” and “khazm;” the same is used for the leaves of dragon’s blood tree in Socotra “sa’af” which is also used for palm leaves. The names in Morocco, Sudan, and Somalia are not linked to Arabic; “ajgal” and “ombet” are in local languages and have been used for the scientific name.

#### Toponym

The use of geographical-/ecological-based toponyms stands as a potentially useful tool for aiding the reconstruction of historical changes. Toponyms have rarely used as a biogeographic indicator of species or vegetation-type occurrences [35]. Analyzing the toponyms (Fig. 5) shows a high frequency of names referring to the terrain. Zeini et al.’s [94] study in Sinai (Egypt) classified 69.9% of their recorded place names as referring to the landforms followed by names that referred to water. Human place names are typically metaphorical, alluding to a resemblance between some physical feature of a site and the shape of the organ after which it is named [13]. In Socotra, human place names describe where things happen and places where people harvest, gather the goats, collect water, or play and they have a general name for a whole area or landmark (like a mountain). Plotting the distribution of plant names in Socotra is another way to appreciate and display the ecological niche and knowledge.

Plant names (phytotoponyms), which are our focus in this research, come in the third position with a frequency of 13.6%. Most of the phytotoponyms are for general names or uses, while 53.7% of all phytotoponyms could be identified by scientific names and 12.2% could not be identified (Table 4). This shows a strong connection between the people and the plants. The identified plants are important for food, fodder, and firewood (*Phoenix*, *Tamarix*, *Punica*), famous for their products such as (*Dracaena* and *Aloe*) [39]. Similarly, Shi et al. [28] mentioned that plant names often used in daily life appear frequently in phytotoponyms. In Socotra, trees and large shrubs easily distinguished in the landscape, such as *Commiphora* and *Maerua*, represent landmarks (Wolf 1998: Camarda 2005 cited by Pinna et al. [37]), and these categories give a good sign for orientation and recognizing the landscape. Water was in the last position, with 5.3% names related to water existence or its amount. Comparing within areas, we found out that eastern sites have more place names related to water than western site, which looks logically correct with eastern Socotra having a higher amount of water than the western areas [42]. Names related to cows and goats represent the main animal toponyms because they are the main livestock on the island. All areas have a similar percentage of names referring to animals that can be explained by the fact that grazing is common in the selected areas and on the island in general. Names with unknown meaning could be linked to ancient language as suggested by Wagner (1960–1964) cited by Pinna et al. [37].

#### Potential implications for past dragon’s blood tree distribution

Dragon’s blood tree name appears 6 times which represent 14.6% of the phytotoponyms, four occurrences in western Socotra with two different variants and two in eastern Socotra with also two different variants. Pérez [95] also noted three different variants of the phytotoponyms “drago” in the Canary Islands, exploring the local dialects. All the names in the western areas of the island are not single names but linked with other words, *D. cinnabari* pool, *D. cinnabari* sign, *D. cinnabari* place, and *D. cinnabari* stand. The six names include two names associated with the existing *D. cinnabari* tree, one name associated with a place close to *D. cinnabari* trees and where there have been trees before, one name associated with a place close to *D. cinnabari* tree but nobody remembers that there was a tree before, one name with no tree near but according to the local people it could exist, and one name with no trees and historically nobody knows of the trees existence on the area.

Half of the *D. cinnabari* place names occur in Qataria, the area with a few limited isolated trees. Qataria is the farthest western site of current *D. cinnabari* distribution on the island, and the place names of *D. cinnabari* are near and around the remnant *D. cinnabari* trees. According to the local people in the area, there is a possibility of a small dragon’s blood trees in the cliffs towards the sea, which provides an opportunity for discovering new *Dracaena* sites in the area. This area is the western end of the ridge stretching from the central Hagher Mts., and it indicates that the entire ridge could be likely covered by *Dracaena* forests in the past, even though Attorre et al. [47] did not assign most of this area to the model of potential *Dracaena cinnabari* distribution. In the second area in Momi, on the eastern side of the island, there is a *D. cinnabari* place name, but according to the local community, there is also the possibility of *D. cinnabari* trees on the cliffs towards the sea. In fact, this area is not far from the recent *Dracaena* population and was included in the potential *Dracaena* occurrence made by Attorre et al. [47].

The third place is Ma’aleh (in the west), where *D. cinnabari* is a place name but no more information is available and there was no opportunity for visiting the site. This finding is the most important because is the furthest from recent *D. cinnabari* distribution. In the entire western part of Socotra, there is no one current record of *Dracaena* occurrence beyond Qataria [54] and this toponym would also confirm the model of potential *Dracaena* occurrence in the Ma’aleh’s highland published by Attorre et al. [47].

The last *D. cinnabari* place name in the Hagher is close to *D. cinnabari* population, but the name is for a place without *D. cinnabari* trees and they do not know the presence of the tree in the place before; another *D. cinnabari* place name was also recorded from Hagher towards To’ahor’s campsite-outside or study area (communication with local people).

In all investigated areas, there are still some preserved plant species accompanying dragon’s blood tree woodlands as *Boswellia ameero*, *B. elongata*, *Buxanthus pedicellatus*, *Commiphora planifrons*, *Euphorbia socotrana*, or *Euryops arabicus* [96–102]. The occurrence of these plants can serve as an indirect indicator of previous *D. cinnabari* distribution according to the associated plant communities.

Generally, the *D. cinnabari* place names seem to be associated with the current and potential distribution. A similar result was obtained by Pérez [95] for *Dracaena draco* on Gran Canaria. In his map, there are 42 phytotoponyms: 2 names associated with existing *D. draco* occurrence and others are close to the current distribution of individual trees or in the area of potential distribution. The population decline of *Dracaena draco* is much larger than thus documented on Socotra Island by many authors [43, 47, 103–106]. Overgrazing destroying the natural regeneration [107] and very slow growth of recruitment [49] do not allow the trees to escape from the browsing zone [50, 54, 108]. Therefore, these are known as the main reasons for the population decline. The loss of each tree leads to a decrease in biodiversity, as dragon’s blood trees are important nurse trees [53] and habitats for animals also [109, 110]. The loss of dragon’s blood trees may also affect the hydrological cycle as these plants capture horizontal precipitation [111].

## Conclusion

In Socotra, luckily, the landscape still has its original characteristic with relatively little human interventions [74], although the natural and human landscape is rapidly changing [112]. Ethnobotanical knowledge has been preserved within its unique language [39]. We can summarize our conclusions in seven main points:

1. *Dracaena cinnabari* toponyms exist in Socotra and seem related to areas where currently no trees are present, yet they were historically a feature of the place; this could support the argument that the distribution of *Dracaena* was larger in the past [47].
2. The *D. cinnabari* trees could be distributed to the whole medium to higher altitude areas on the island, potentially from the west in Ma’aleh to the east in Momi, before humans inhabited the island.
3. Currently, the Arabic common name for dragon’s blood resin is “dum al-akhawin” and that for the tree is “ahrieb” and can be generalized.
4. The local name that appeared in history for the main product is “eda’a” (mixed-cooked dragon’s blood), and this can be a sign that “eda’a” was the main product exported from Socotra.
5. There could be potentially new areas for finding *D. cinnabari* trees especially in the cliff areas towards the sea in Qataria and Momi, also the northeastern slopes of Hagher towards Momi plateau in the east, and Noged plain to the North.
6. This result can be an important part of conservation efforts, and those areas with *D. cinnabari* toponyms could be potential areas for future reforestation of this species, where ecological conditions allow.
7. The study has stressed the need for documenting place names and knowledge related as part of preserving the cultural heritage related to plants of the Socotra Archipelago and the importance of using this knowledge for sustainable resource management. This study is just a first step for further use of toponyms and can be repeated for other important species or historical land use.

## Data Availability

All data generated or analyzed during this study are included in this published article (and its supplementary information files).
